# Eye Injuries Sustained at a Foam Party — Collier County, Florida 2012

**Published:** 2013-08-23

**Authors:** Philip P. Cavicchia, Sharon Watkins, Carina Blackmore, James Matthias

**Affiliations:** Florida Dept of Health; CDC/Council of State and Territorial Epidemiologists Applied Epidemiology Fellow

On May 26, 2012, the Collier County Health Department was notified by law enforcement and hospital personnel that approximately 40 persons had sought care at local emergency departments because of severe eye irritation and pain. Patients reported that they had attended a foam party at a local nightclub the night before. Syndromic surveillance activities carried out by the Florida Department of Health identified 35 patients who had visited an emergency department in Collier County on May 26 with a chief complaint related to eye injuries, well above the expected number of less than 10.

At foam parties, soapy foam is sprayed onto the dance floor while participants dance. The foam is distributed from blowers on the ground or attached to the ceiling, and several feet of foam can accumulate. Foam parties can last for several hours while foam is dispersed intermittently throughout the night. Products used at these events to produce foam contain ingredients similar to those in soaps and shampoos, such as sodium lauryl sulfate. Some formulations used at foam parties are proprietary, and chemicals, chemical compositions, and concentrations are unknown. For use at a foam party, the product is purchased in a highly concentrated form and diluted with water before use. This was the third foam party of the year at this nightclub.

An investigation was initiated by the Florida Department of Health to determine the extent and severity of the injuries. A case was defined as an eye injury (self-reported or identified using specific codes in the *International Classification of Diseases, Ninth Revision* [ICD-9] during medical record abstraction) in a person who had attended a specific local nightclub foam party in Naples, Florida, from the evening of May 25 through the morning of May 26, 2012. Efforts to identify cases involved abstraction of medical records from patients treated in emergency departments at the two major hospital systems in Collier County. Records were requested for any person who presented with symptoms of eye irritation or chemical burns during May 25–June 1, 2012. Medical records also were requested, using specific ICD-9 codes, from ophthalmology clinics, urgent-care centers, and the neighboring county hospital system where patients were known to have sought care. Using contact information obtained from medical records, patients were contacted and interviewed over the telephone. An incident-specific questionnaire was developed to obtain information on demographics, foam party attendance, foam exposures, potential risk factors, symptoms and injuries, medical care received, and previous foam party experiences. Additional attendees were identified by asking interviewees if they had attended the party with another person, and if so, were they willing to provide the contact information for them.

Medical record abstractions in Collier County hospitals identified 30 cases of injury related to the foam party. Interviews, contacts provided by local law offices, and additional medical record abstractions from ophthalmology clinics, urgent-care centers, and neighboring county hospitals led to the identification of an additional 26 cases. A total of 56 persons meeting the case definition were identified during the investigation out of approximately 350 persons thought to have attended the party. Thus, an estimated 16% of attendees suffered eye injuries as a result of this event, and 43 (76.8%) of them received medical care.

In all cases, injured persons reported getting foam in their face, with 96% (n = 44) of interviewed persons reporting eye exposure. Almost 90% of interviewed persons reported rubbing their eyes after exposure to the foam. Eye irritation (94.6%), severe eye pain (91.1%), pink eye/redness (87.5%), decreased visual acuity (81.3%), and conjunctivitis (76.8%) were the most common injuries (Table). Of note, half of the cases were diagnosed with abrasions of the cornea (n = 28). For those persons who sought medical care, the average number of visits was 3.2. In 11 cases, patient’s visual acuity could not be tested in at least one eye during their initial medical-care visit because they were unable to open their eye or read the first letter of the chart. Among persons interviewed, the average duration of symptoms was 7 days (median: 6 days), ranging from less than 1 hour to more than 1 month. In seven cases, symptoms had not completely resolved at the time of the interview (i.e., more than 1 month after the injury).

Although some persons experienced minor eye irritations related to foam exposure, many experienced more serious eye injuries, such as decreased visual acuity (n = 39), conjunctivitis (n = 43), and corneal abrasions (n = 28). This investigation highlights the range and potential seriousness of eye injuries that can result from exposure to foam.

## Figures and Tables

**TABLE:** Number and percentage of persons with injuries and symptoms resulting from a foam party at a nightclub — Collier County, Florida, May 2012

Characteristic	No.	(%)
**Received medical care**		
No	13	(23.2)
Yes	43	(76.8)
**Eye injuries**	**56**	**(100.0)**
Eye irritation	53	(94.6)
Severe eye pain	51	(91.1)
Pink eye/Redness	49	(87.5)
Decreased visual acuity†	39	(81.3)
Conjunctivitis	43	(76.8)
Photophobia	40	(71.4)
Drainage	32	(57.1)
Abnormal pH	28	(50.0)
Corneal abrasions	28	(50.0)
Tearing§	17	(40.5)
Blurry vision§	16	(38.1)
Watery discharge§	9	(21.4)
Foreign body sensation	2	(3.6)
**Non–eye-related injuries**	**18**	**(32.1)**
Skin irritation/Rash	14	(25.0)
Slips/Falls	3	(5.4)
Allergic reaction	2	(3.6)

* N = 56.

† Because of missing information for eight cases, n = 48.

§ Medical record only (n = 42).

